# Identification and Morphological Characterization of Biofilms Formed by Strains Causing Infection in Orthopedic Implants

**DOI:** 10.3390/pathogens9080649

**Published:** 2020-08-12

**Authors:** Débora C. Coraça-Huber, Lisa Kreidl, Stephan Steixner, Maximilian Hinz, Dietmar Dammerer, Manfred Fille

**Affiliations:** 1 Research Laboratory for Biofilms and Implant Associated Infections (BIOFILM LAB), Experimental Orthopedics, Department of Orthopedic Surgery, Medical University of Innsbruck, Peter-Mayr-Strasse 4b, Room 204, 6020 Innsbruck, Austria; lisa.kreidl@student.i-med.ac.at (L.K.); stephan.steixner@i-med.ac.at (S.S.); maximilian.hinz@student.i-med.ac.at (M.H.); 2 Department of Orthopedic Surgery, Medical University of Innsbruck, Anichstraße 35, A-6020 Innsbruck, Austria; dietmar.dammerer@i-med.ac.at; 3 Institute of Hygiene and Medical Microbiology, Medical University Innsbruck, Schöpfstrasse 41, 6020 Innsbruck, Austria; manfred.fille@i-med.ac.at

**Keywords:** biofilm, in vitro conditions, antibiotic susceptibility, implant-related infections

## Abstract

**Objectives**: For a better understanding of the mechanisms involved in biofilm formation, we performed a broad identification and characterization of the strains affecting implants by evaluating the morphology of biofilms formed in vitro in correlation with tests of the strains’ antibiotic susceptibility in planktonic form. The ability of the strains to form biofilms in vitro was evaluated by means of colony forming units counting, metabolic activity tests of biofilm cells, and scanning electron microscopy. **Methods**: A total of 140 strains were isolated from patients with orthopedic implant-related infections during the period of 2015 to 2018. The identification of the isolates was carried out through microbiological cultures and confirmed by matrix-assisted laser desorption/ionization time-of-flight mass spectrometry. Antibiotic susceptibility rates of the isolates were accessed according to EUCAST (European Committee on Antimicrobial Susceptibility Testing). The ability of all isolates to form biofilms in vitro was evaluated by counting the colony forming units, by measuring the metabolic activity of biofilm cells, and by analyzing the morphology of the formed biofilms using scanning electron microscopy. **Results**: From all the isolates, 41.84% (62 strains) were *Staphylococcus epidermidis* and 15.60% (22 strains) were *Staphylococcus aureus*. A significant difference in the capacity of biofilm formation was observed among the isolates. When correlating the biofilm forming capacity of the isolates to their antibiotic susceptibility rates, we observed that not all strains that were classified as resistant were biofilm producers in vitro. In other words, bacteria that are not good biofilm formers can show increased tolerance to multiple antibiotic substances. **Conclusion**: From 2015 until 2018, *Staphylococcus epidermidis* was the strain that caused most of the orthopedic implant-related infections in our hospital. Not all strains causing infection in orthopedic implants are able to form biofilms under in vitro conditions. Differences were observed in the number of cells and morphology of the biofilms. In addition, antibiotic resistance is not directly related to the capacity of the strains to form biofilms in vitro. Further studies should consider the use of in vitro culture conditions that better reproduce the joint environment and the growth of biofilms in humans.

## 1. Introduction

Around two thirds of all human infections are believed to be biofilm related. Biofilm-associated implant-related bone and joint infections are clinically important due to the extensive morbidity, cost of care, and socioeconomic burden that they cause [1,2,3]. The predominantly isolated bacteria from implant infections in the orthopedic area are usually part of the physiological skin microflora. *Staphylococcus aureus*, *Staphylococcus epidermidis*, coagulase-negative *staphylococci*, and *enterococci* are the microorganism usually involved in such cases. A biofilm can be described as a complex and well-structured aggregation of microorganisms of one or more species. Biofilms are found adherent to biotic (host tissue) and abiotic (implant/biomaterial) surfaces or as floating aggregates, all of which are encased in a self-produced matrix of polymeric substances [4]. Biofilm formation is also related to increased bacterial antibiotic resistance and/or tolerance. In the strictest sense, multidrug-resistant organisms (MDROs) are labelled as such because of their in vitro resistance to more than one antimicrobial agent. Infections with MDROs can lead to inadequate or delayed antimicrobial therapy and are associated with poorer patient outcomes [5,6,7,8]. Biofilm-specific antibiotic resistance and tolerance mechanisms are multifactorial, varying depending on the particular antimicrobial agent; the bacterial strain and species; the age and developmental stage of the biofilm; and the biofilm growth conditions [9,10,11,12,13]. Individually, no single mechanism can account for the heightened antibiotic recalcitrance that is characteristic of biofilms. In combination, however, these resistance and tolerance mechanisms severely limit the ability to effectively treat biofilm-based infections with the antimicrobial arsenal that is currently available [14].

A variety of methods can be used to establish biofilm models in a laboratory. Each culture method has its own unique advantages and disadvantages depending on the experimental question that is being asked. These culture methods, which are very well described elsewhere, include, among many others, biofilms grown on multi-well plates, biofilms grown on pegs, colony biofilms, biofilms cultured in flow cells, biofilms grown in continuous flow tube reactors, drip-fed biofilms, biofilms grown on coupons in rotating disk reactors, and in vivo biofilm models [14,15,16,17,18,19,20,21,22]. During the development of a biofilm, different morphology can be observed among different species and over different incubation times. In addition, the literature suggests that species-specific factors determine the architecture, morphology, and size of the biofilms [23].

An evaluation of several different isolates, comparing their biofilm forming ability, morphology, and rate of growth, is still missing in the literature. For a better understanding of the mechanisms involved in biofilm formation, we carried out a broad identification and characterization of the strains affecting implants, evaluating the morphology of biofilms formed in vitro in correlation with tests of the strains’ antibiotic susceptibility in planktonic form. The ability of the strains to form biofilms in vitro was evaluated by means of colony forming units counting, metabolic activity tests of biofilm cells, and scanning electron microscopy.

## 2. Material and Methods

### 2.1. Isolation and Identification of the Strains

For this study, 140 patient-isolated strains were evaluated. We obtained the strains during the period of 2015 until 2018 from patients undergoing prosthetic joint infection (PJI) treatment at the Department of Orthopedic Surgery of the Medical University Innsbruck, Austria. Tests were carried out only with bacterial strains, which would otherwise be discarded from the routine lab from our institution. The protocol used in this study was evaluated and approved by the Human Ethics Committee of the Medical University Innsbruck (AN2017-0072 371/4.24 396/5.11-4361A). We carried out the identification of all isolates using conventional microbiological cultures followed by confirmation of bacterial identification using matrix-assisted laser desorption/ionization time-of-flight mass spectrometry (MALDI-TOF-MS) [24,25,26]. The Division of Hygiene and Medical Microbiology Department of the Medical University Innsbruck carried out the identification of bacterial strains using MALDI-TOF technology under certification ISO EN 9001-2008. After identification, each strain was cryopreserved at −80 °C in special medium until the realization of the tests.

### 2.2. Antibiotic Sensitivity Tests

For the obtainment of antibiotic susceptibility rates, the isolated strains were suspended in saline solution (0.85% NaCl w/v in water) at a McFarland of 0.5 (1–2 × 10^8^ CFU/mL). Using a sterile cotton swab, the solutions were inoculated on different agar mediums according to the strain species: *Staphylococci* were inoculated on Mueller-Hinton agar; all the others were inoculated on Mueller-Hinton agar enriched with 5% horse blood and 20 mg/L ß-NAD (β-nicotinamid adenin dinucleotide) (bioMérieux Austria GmbH, Vienna, Austria). After 15 min of inoculation, the antibiotic discs (BBL™ Sensi-Disc™ Susceptibility Test Discs, BD Life Sciences, Heidelberg, Germany) were carefully placed on agar plates. Using standard protocols, 30 different antibiotics were tested. For inpatient care cases or specific strains, we used additional antibiotics. The list of all antibiotics used and their concentrations can be found in the supplementary material (Table S1). After placing the antibiotic discs in the agar plates, the plates were incubated at 37 °C for 16–20 h. After incubation, the zones of inhibition were measured, and the size of the zones, based on millimeter measurements, were converted into categories of susceptibility (susceptible, intermediate, and resistant) according to EUCAST (European Committee on Antimicrobial Susceptibility Testing) [27]. The Division of Hygiene and Medical Microbiology Department of the Medical University Innsbruck carried out the antibiotic susceptibility tests under certification ISO EN 9001-2008.

### 2.3. Biofilm Formation In Vitro

For the biofilm formation, conventional 96-well plates (Corning®, Amsterdam, The Netherlands) were used. Three colonies of each strain were suspended in 2 mL tryptic soy broth enriched with 1% glucose (TSB-G) in a 15 mL centrifuge tube (VWR International, Radnor, PA, USA) and incubated at 37 °C in a moist chamber under constant circular shaking (Edmund Bühler GmbH, Hechingen, Germany) at 200 rpm for 24 h. After incubation, the bacterial solutions were diluted in fresh TSB-G to a 10^5^ bacteria/mL concentration. Afterwards, 100 µL of the diluted bacterial solutions were added in the wells, each containing a sterile stainless-steel disc (DIN9021, stainless steel A2, size M2, diameter 5.9 mm) as the substrate for biofilm growth. The 96-well plates were incubated in a moist chamber under constant circular shaking at 37 °C for 48 h for the obtainment of biofilms. These experiments were carried out in triplicate.

### 2.4. Counting of Colony Forming Units (CFU)

After incubation, the discs containing biofilms were removed from the media, washed in phosphate-buffered saline solution (PBS, pH 7.4) for removal of planktonic cells, and added to tubes containing fresh PBS. The discs were sonicated for 3 min at high intensity (Bactosonic, Bandelin electronic GmbH & Co. KG, Berlin, Germany). The sonication fluid was transferred to a Mueller-Hinton agar plate (50 µL) with an automatic spiral plater (model WASP 2, Don Whitley Scientific, Shipley, UK). The plates were incubated at 37 °C. After the incubation, the colony forming units (CFU) were counted. The tests were carried out in duplicate.

### 2.5. Metabolic Activity Tests

The metabolic activity of the biofilm cells was characterized as high (OD490 ≥ 0.1), low (0.025 ≤ OD490 < 0.1), and no activity (OD490 < 0.025) [28]. Metabolic activity tests were carried out directly on the biofilms formed by each strain using the colorimetric method. The amount of reduced tetrazolium salt (3-Bis-(2-Methoxy-4-Nitro-5-Sulfophenyl)-2H-Tetrazolium-5-Carboxanilide; XTT) was quantified using a spectrophotometer (450 nm wavelength). After 48 h of incubation and obtainment of the biofilms, supernatants were aspirated, and the discs were transferred to a new 96-well plate and washed three times with 200 µl of PBS. Then, 100 µl of PBS plus 50 µl of the final XTT solution were added to each well. Plates were incubated for three hours at 37 °C, protected from light, and constantly stirred with circular shaking. After incubation, 100 µL of the supernatant containing the reaction mixture were added to a 96-well plate and analyzed with a spectrophotometer (Multiskan Fc, Thermo Fisher Scientific, Shanghai, China).

### 2.6. Scanning Electron Microscopy

After biofilm growth, the discs were removed from media, washed for removal of planktonic cells, and immersed in 1 mL of glutaraldehyde 2.5% for fixation. After fixating for 24 h at 4 °C, the discs were dehydrated with an ascending alcohol series (50%, 70%, 80%, then 99.9% ethanol). After the last step, the discs were placed in an incubator to dry out. The dried discs were placed on aluminum pins and fixed with Leit-C (Göcke, Plano GmbH, Wetzlar, Germany). The pins were sputtered with Au (Agar Sputter Coater, Agar Scientific Ltd., Stansted, GB, UK) for 1 min and analyzed by scanning electron microscopy (SEM, JSM-6010LV, JEOL GmbH, Freising, Germany).

### 2.7. Characterization of Biofilm Forming Capacity

The characterization of biofilm forming capacity from each strain was evaluated by counting the CFU and by analyzing the biofilms’ morphology using scanning electron microscopy. According to the literature, biofilms are typically characterized by dense, highly hydrated clusters of bacterial cells. These cells secrete extracellular polymeric substances that hold the cell aggregates together. In some cases, clusters of cells are separated by channels through which fluid can move [23,29,30,31]. In this study, a classification was given for each strain for the biofilm formation capacity based on these two methods. The strains that showed high numbers of CFU (10^5^–10^9^) and those that formed massive-to-moderate biofilms with three-dimensional (3D) architecture, slime-like substances covering the bacteria, as well as fluid channels within the bacteria aggregates were scored as (HIGH); the strains that showed low numbers of CFU (10^1^–10^4^) and that formed a few isolated biofilms without 3D structures were scored as (LOW); and the strains that showed no CFU as well as no biofilm formation were scored as (NO BIOFILM) [32,33]. 

### 2.8. Data Analysis

The results were evaluated by using GraphPad Prism 7.00 (GraphPad Software, Inc., La Jolla, CA, USA) and the Statistical Package for the Social Sciences (SPSS®; IBM Computer Hardware Company; New York, NY, USA). The results obtained from the CFU counting, XTT tests, and antibiotic sensitivity tests were analyzed by Pearson chi-square for distribution of the data among the tests. Significant differences were considered as *p* values of < 0.005. The Pearson chi-square test was carried out only with *S. epidermidis* as this strain was the one isolated most often from our patients. The null hypothesis of this study was that there is no correlation between the antibiotic resistance profile and the biofilm forming ability of the isolated strains.

## 3. Results and Discussions

**Figure 1:**
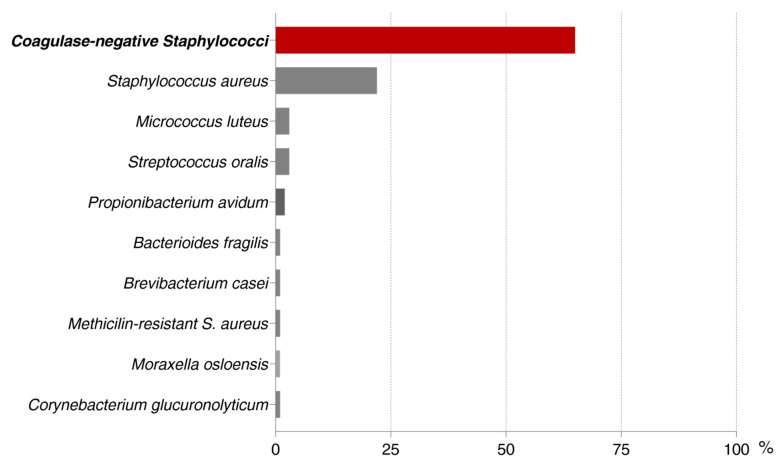
Clinical isolates obtained during the period of 2015 until 2018 from patients undergoing prosthetic joint infection (PJI) treatment.

In total, 140 isolated strains were selected for this study. All the bacterial strains included in this study are described in the Figure 1**.** From all the strains, *Staphylococcus epidermidis* predominated with 41.84% (62 strains), *Staphylococcus aureus* with 15.60% (22 strains), and the rest divided between coagulase-negative *Staphylococci* and other strains (Figure 1). A great variation in the capacity of forming biofilms in vitro was observed between the bacterial species. The differences in the intensity of biofilm formation for all strains are displayed in the Table 1. 

The phenotypic switch from a free-swimming, planktonic lifestyle to a sessile existence in a biofilm is a highly regulated developmental process that depends on numerous environmental and genetic factors, which vary from species to species [14,34,35,36,37,38,39]. The influence of the host environment on the biofilm formation is a subject of investigation. Elements such as proteins and ions and stress factors are directly related to the ability of bacteria to aggregate and form a biofilm [40,41,42,43,44]. The biofilm in vitro model used in this study is well established and has been used by several other authors for studying biofilm formation mechanisms and for susceptibility tests using substances and surfaces. A question that arose during the present study was if the biofilm model used is sufficient to reproduce the biofilm formation present on the implant surfaces and interfaces with host tissues. According to Xu, W. and collaborators (2017), in vitro assays for analysis of biofilm formation and growth are exquisitely sensitive to medium conditions, and it is not always clear that the conditions established for optimal formation of biofilm in vitro are representative of the in vivo environment encountered by strains during pathogenesis [40]. In our opinion, media that simulate the joint environment may be more suitable for studying orthopedic implant-related biofilms in vitro.

### 3.1. Colony Forming Units Counting

The average of CFU formation showed different rates of biofilm growth between all the strains tested. From all *S. epidermdis* strains tested, only one showed no CFU formation. The rest showed a great variation in growth intensity (Table 2 and Table 3). The same was observed for the *S. aureus* strains as well as for all other strains tested.

### 3.2. Metabolic Activity of Biofilm Cells

The metabolic activity varied among the biofilms formed by each isolate. Some *S. epidermidis* biofilms showed weak activity while several strains formed biofilms with high metabolic activity (Table 3 and Table 4). Almost half of the isolates presented biofilms with very slight or no detectable activity at all. The same was observed between the *S. aureus* strains, where almost 50% of the evaluated strains formed biofilms with high-to-low activity and the rest very slight or no detectable metabolic activity. The same variation was observed in the other evaluated strains. Biofilm formation is a protection mechanism developed by microorganisms to survive in stressful environments [45]. In each patient, the surroundings of an implant offer a different environment and conditions for biofilm formation. Not all patients present the same clinical status and some differences, for example, in immunity and physiology, could interfere in the development of a biofilm. The environmental conditions and physiological responses of the bacteria to their local environment are not homogeneous throughout a biofilm. The metabolic activities of the cells, together with diffusional processes, result in concentration gradients of nutrients, signaling compounds, and bacterial waste within biofilms. As the bacteria respond to these gradients, they adapt to the local chemical conditions, which can change over time and be physiologically distinct from planktonic cells, but also vary from each other, both spatially and temporally, as biofilm development proceeds [29]. The results of metabolic activity were not taken into consideration for the biofilm formation capacity classification because of the hypothesis that a highly developed biofilm presents less metabolic activity because its cells are in a dormant phase.

### 3.3. Scanning Electron Microscopy of the Biofilms

**Figure 2:**
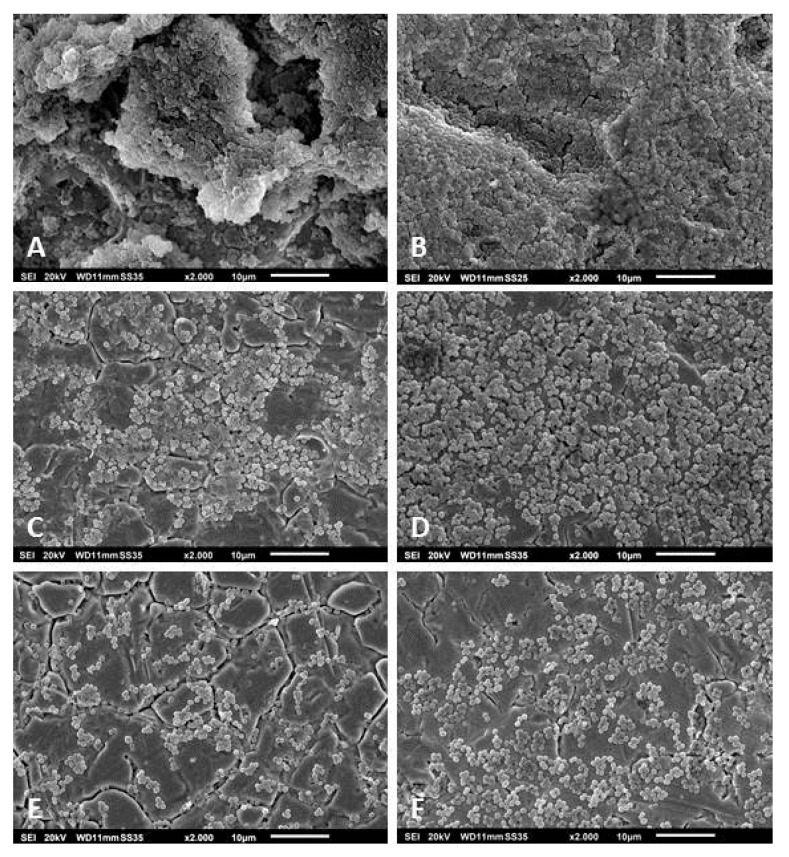
Scanning electron microscopy of *S. epidermidis* biofilms isolated from patients undergoing PJI treatment. The images show massive (**A**,**B**), moderate (**C**,**D**), and slight (**E**,**F**) biofilm formation. Magnification for all images: 2000×. Specimens were analyzed by scanning electron microscopy (SEM, JSM-6010LV, JEOL GmbH, Freising, Germany).

**Figure 3:**
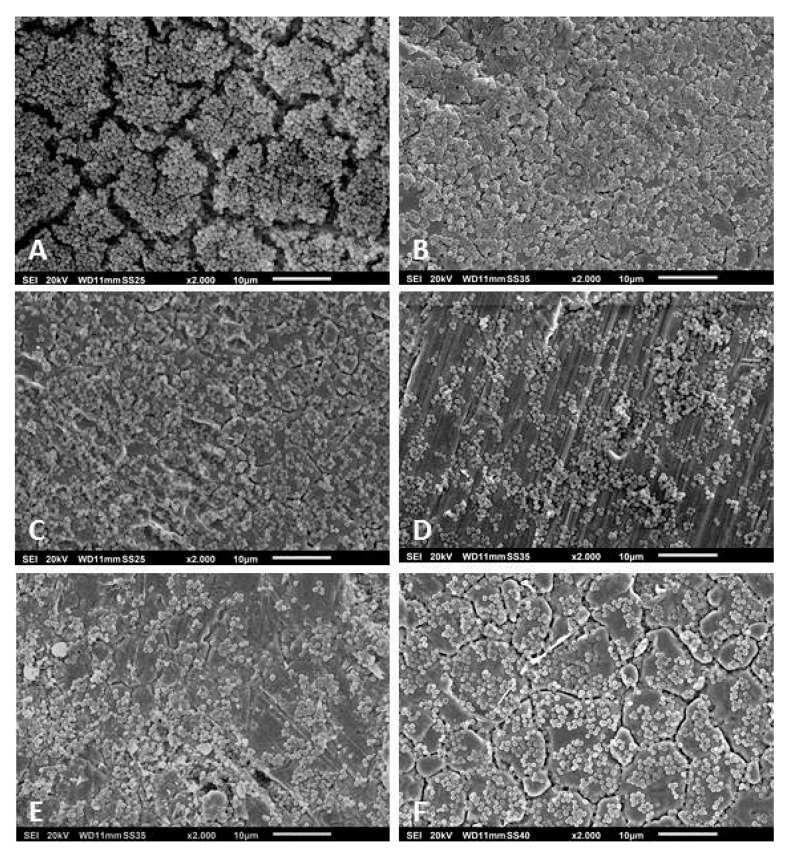
Scanning electron microscopy of *S. aureus* biofilms isolated from patients undergoing PJI treatment. The images show massive (**A**,**B**), moderate (**C**,**D**), and slight (**E**,**F**) biofilm formation. Magnification for all images: 2000×. Specimens were analyzed by scanning electron microscopy (SEM, JSM-6010LV, JEOL GmbH, Freising, Germany).

**Figure 4:**
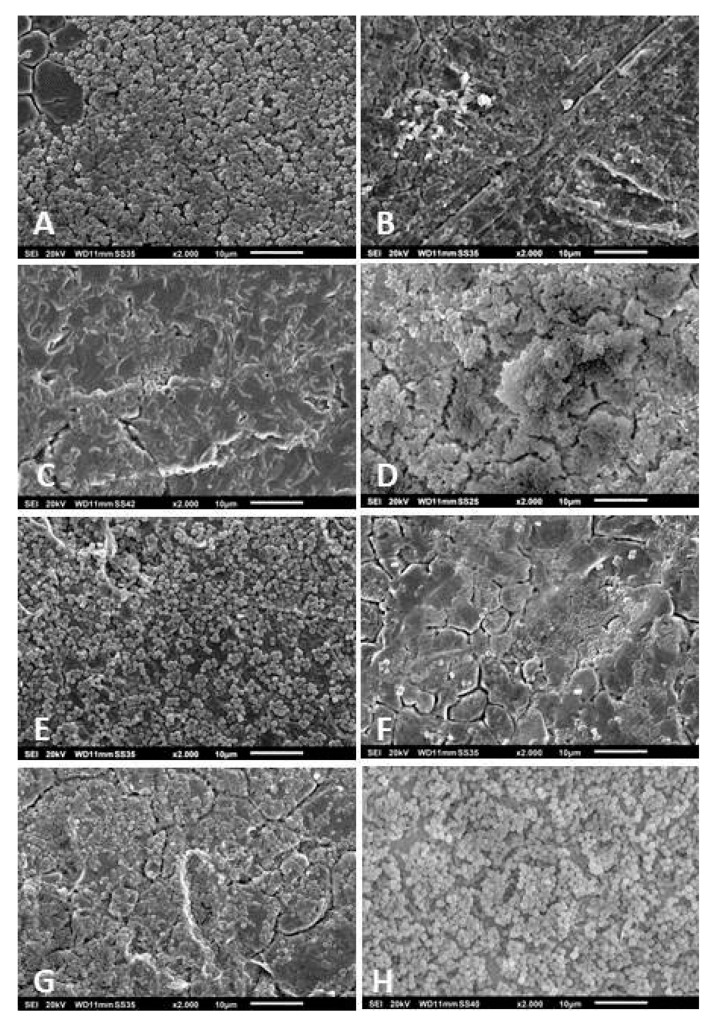
Scanning electron microscopy of *S. haemolyticus* (**A**); *S. oralis* (**B**); *P. avidum* (**C**); *S. lugdunensis* (**D**); *S. saprophyticus* (**E**); *B. casei* (**F**); *S. hominis* (**G**); and *S. capitis* (**H**) biofilms isolated from patients undergoing PJI treatment. Magnification for all images: 2000×. Specimens were analyzed by scanning electron microscopy (SEM, JSM-6010LV, JEOL GmbH, Freising, Germany).

**Figure 5:**
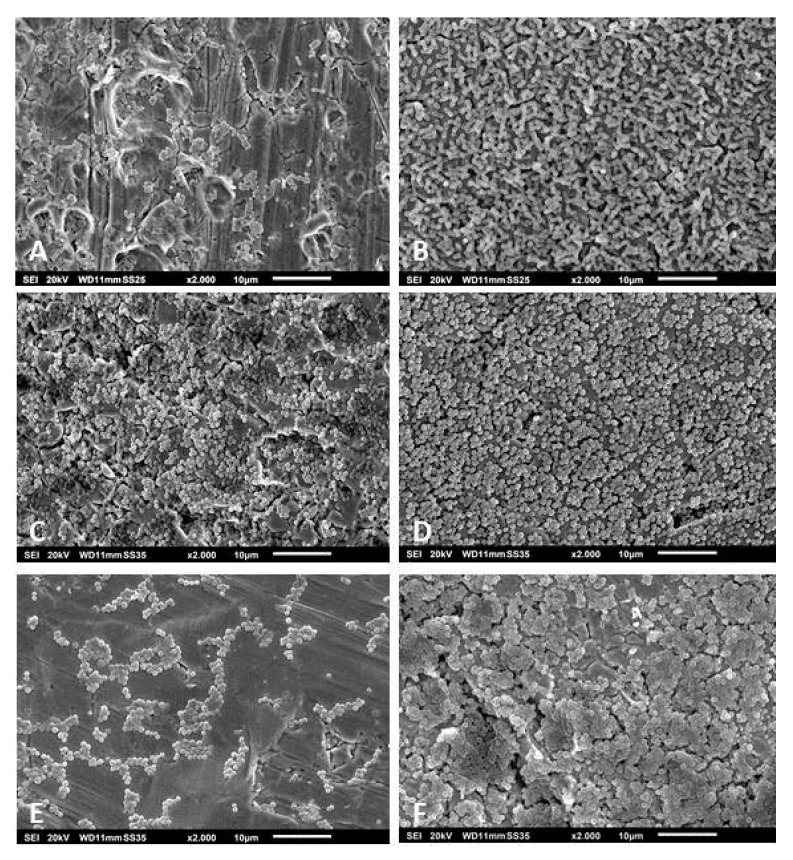
Scanning electron microscopy of *Corynebacterium glucuronolyticum* (**A**); *M. osloensis* (**B**); MRSA (**C**); *S. cohnii* (**D**); *S. simulans* (**E**); and *S. xylosus* (**F**) biofilms isolated from patients undergoing PJI treatment. Magnification for all images: 2000×. Specimens were analyzed by scanning electron microscopy (SEM, JSM-6010LV, JEOL GmbH, Freising, Germany).

After cultivating the strains in vitro for 48 h, we observed that not all were able to form three-dimensional and slime-rich biofilm structures. Of the 62 *S. epidermidis* strains, 12 did not form biofilms in vitro. The other 50 strains formed slight-to-massive biofilms. The high biofilm formation showed mushroom-like structures, fluid channels, and slime-like substance covering the cell aggregates (Figure 2A). Some strains formed massive biofilms covering a significant area on the surface of the metal disc (detail on Figure 2B). The *S. epidermidis* strains that presented low biofilm forming capacity showed no mushroom-like structures and slime was only observed partially covering the bacterial aggregates (Figure 2C–F). From the 22 *S. aureus* isolates, four were not able to form biofilms. The other isolates formed biofilms that variated from low-to-high formations. The high formation showed slimy mushroom-like structures covering the disc’s surface (Figure 3A,B). In both the moderate and low biofilm formations, some islands of biofilms were observed between cells covering the metal’s surface (Figure 3C–F). The remaining isolates, *B. fragilis*, *M. luteus*, *S. caprae*, *S. pettenkoferi,* and *S. warneri* were not able to form biofilms in vitro. *S. haemolyticus* presented high biofilm forming capacity with a biofilm covered with a slime-like substance (Figure 4A). *S. oralis* formed a moderate biofilm where some cell aggregates were observed between isolated cells attached on the metal surface (Figure 4B). *P. avidum* formed a moderate to massive biofilm with less of a slime-like substance and no three-dimensional or mushroom-like structures (Figure 4C). *S. lugdunensis* formed massive biofilms covered with slimy substances and forming three-dimensional structures (Figure 4D). *S. saprophyticus* presented low-to-high biofilm formation with no slimy substances nor three-dimensional structures (Figure 4E). The same was observed with the *B. casei* isolates (Figure 4F). *S. hominis* and *S. capitis* followed the same characteristics of some *S. epidermidis*, where a biofilm was formed with fewer slime-like substances. Three-dimensional structures were observed mostly on *S. capitis* strains (Figure 4G,H). The remaining strains also showed variation in the biofilm forming capacity. *C. glucuronolyticum* and *S. simulans* presented low biofilm forming capacity where islands of cells could be observed dispersed on the metal disc surface (Figure 5A,E), while *M. osloensis*, MRSA, *S. cohnii*, and *S. xylosus* formed massive biofilms covering the entire surface of the discs and showing three-dimensional structures as well slime-like substances covering the cells (Figure 4B–D,F). 

**Figure 6:**
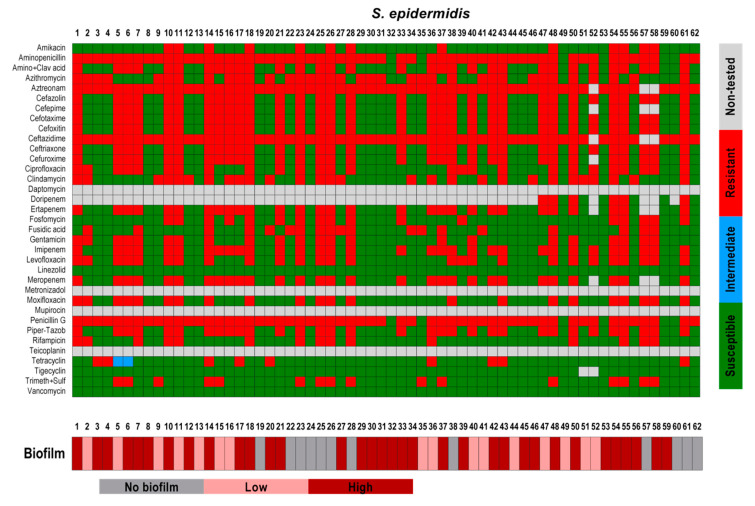
Antibiotic susceptibility of *S. epidermidis* strains in relation to its biofilm forming capacity. The results of the susceptibility tests were classified as susceptible (**green**), intermediate (**blue**), and resistant (**red**) according to EUCAST (European Committee on Antimicrobial Susceptibility Testing). Non-tested antibiotics are displayed in **grey**. The biofilm forming capacity was classified as no biofilm former (**grey**), low biofilm former (**rose**), and high biofilm former (**dark red**). The biofilm formation was classified according to the counting of colony forming units; measurement of metabolic activity; and morphology of the biofilms based on the scanning electron microscopy analysis.

**Figure 7:**
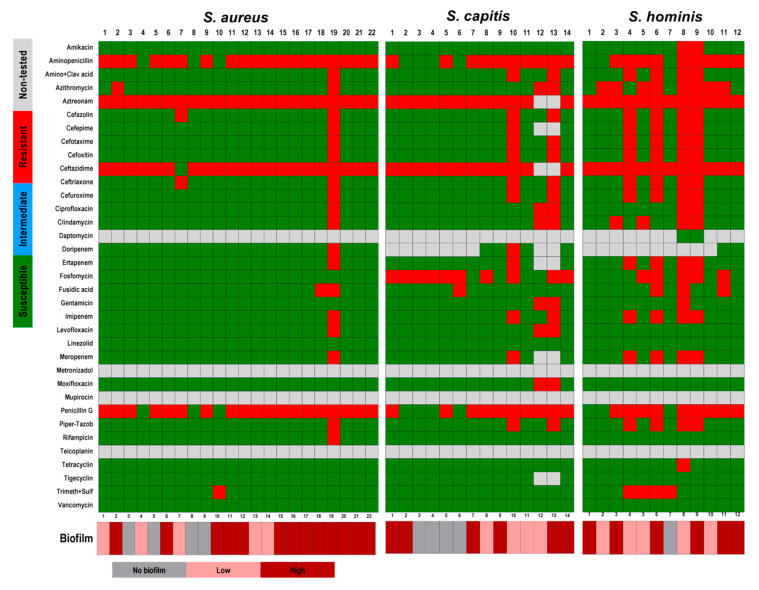
Antibiotic susceptibility of *S. aureus*, *S. capitis*, and *S. hominis* strains in relation to their biofilm forming capacity. The results of the susceptibility tests were classified as susceptible (**green**), intermediate (**blue**), and resistant (**red**) according to EUCAST (European Committee on Antimicrobial Susceptibility Testing). Non-tested antibiotics are displayed in **grey**. The biofilm forming capacity was classified as no biofilm former (**grey**), low biofilm former (**rose**), and high biofilm former (**dark red**). The biofilm formation was classified according to the counting of colony forming units; measurement of metabolic activity; and morphology of the biofilms based on the scanning electron microscopy analysis.

**Figure 8:**
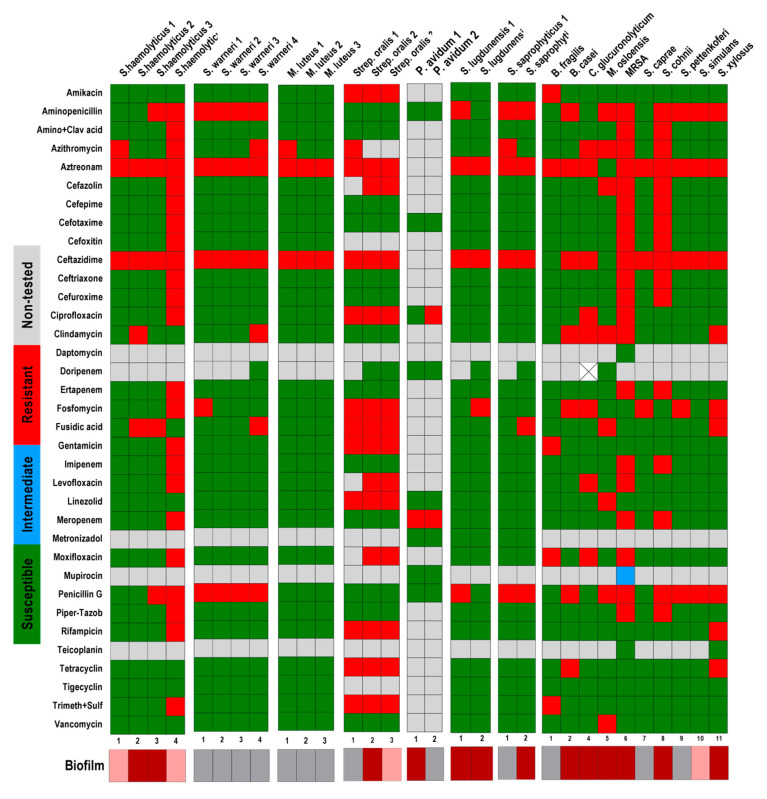
Antibiotic susceptibility of *S. haemolyticus*, *S. warneri*, *M. luteus*, *S. oralis*, *P. avidum*, *S. lugdunensis*, *S. saprophyticus*, *B. fragilis*, *B. casei*, *C. glucuronolyticum*, *M. osloensis*, MRSA, *S. caprae*, *S. cohnii*, *S. pettenkoferi*, *S. simulans*, and *S. xylosus* strains in relation to their biofilm forming capacity. The results of the susceptibility tests were classified as susceptible (**green**), intermediate (**blue**), and resistant (**red**) according to EUCAST (European Committee on Antimicrobial Susceptibility Testing). Non-tested antibiotics are displayed in **grey**. The biofilm forming capacity was classified as no biofilm former (**grey**), low biofilm former (**rose**), and high biofilm former (**dark red**). The biofilm formation was classified according to the counting of colony forming units; measurement of metabolic activity; and morphology of the biofilms based on the scanning electron microscopy analysis.

In addition to the CFU counting, metabolic activity, and morphological analysis of the biofilms, we observed if a relation between antibiotic resistance and biofilm forming capacity exists between the isolates tested. From all the 140 isolated strains, only 9 were not classified as multidrug-resistant (MDR) organisms [46]. Here, we observed that not all MDR strains are good biofilm producers. In the present study, among the 62 *S. epidermidis* strains that were tested, 30 strains showed resistance to at least 20–25 of the 30 antibiotic substances tested. From those 30 *S. epidermidis* strains, only 10 were able to form massive biofilms in vitro and 8 strains were not able to form any biofilms (Figure 6; Table 2 and Table 4). Among all the 22 *S. aureus* strains, only one strain was resistant to more than 20 antibiotic substances. This strain showed a high biofilm formation rate. The other strains, which were resistant to only four or five antibiotic substances, showed a variation in biofilm forming capacity from non-biofilm formers to high biofilm formers. *S. capitis* strains showed weak biofilm forming capacity, and from the three strains that showed multidrug-resistance, one formed a biofilm only once, and two formed only very small biofilms. The same could be observed with the *S. hominis* strains (Figure 7). The remaining strains showed similar results; *P avidum,* which was susceptible to almost all tested substances, formed massive biofilms while *S. oralis*, which showed more resistance, showed no-to-high biofilm formation capacity. Only MRSA was able to highly form biofilms and also showed resistance to multiple antibiotic substances (Figure 8).

Some studies have shown the relation between biofilm formation and susceptibility to antibiotic substances [31,47,48,49,50,51]. In this study, we observed that strains that are resistant to multiple antibiotic substances were not good biofilm producers in vitro. Here, we observed that strains resistant only to a few antibiotic substances were able to form massive biofilms. In contrast, multiple antibiotics resistant strains did not show biofilm forming capacity in our study. One hypothesis is that strains already equipped with resistance mechanisms do not express genes for biofilm formation. Al-Ahmad, A. and collaborators (2014) also observed in their study that most isolates with markedly high minimal inhibitory concentration values were also either moderate biofilm producers (*E. faecalis*, *Lactobacilli* and *Prevotella buccae*) or high biofilm producers (*Actinomyces spp*, *Streptococcus mutans*, and *Pseudoramibacter alactolyticus*) [52].

Our results showed that some patient-isolated strains that are resistant to multiple antibiotic substances build weak or no biofilms in in vitro conditions. The opposite was observed on strains completely susceptible to antibiotics, which were able to form massive biofilms. Similar results were found by Qi and collaborators [53]. These authors tested the relationship between antibiotic resistance and biofilm formation in *Acinetobacter baumannii* and detected that antibiotic-susceptible isolates tended to form stronger biofilms than those of resistant strains. In addition, strong and weak biofilms provided similar levels of enhancement in antibiotic resistance. These findings raise questions regarding the mechanisms through which bacteria maintain a balance between biofilm formation capacity and antibiotic resistance, as well as how resistant strains achieve high levels of biofilm-specific resistance despite producing weak biofilms [53]. 

## 4. Conclusions

From 2015 until 2018, *Staphylococcus epidermidis* was the strain that caused most of the orthopedic implant-related infections in our hospital. Not all strains causing infection in orthopedic implants are able to form biofilms under in vitro conditions. In our opinion, media that simulate the joint environment may be more suitable for studying orthopedic implant-related biofilms in vitro. Differences were observed in the number of cells and morphology of the biofilms. In addition, the antibiotic resistance rate results were not directly related to the capacity of the strains for forming biofilms in vitro. Further studies should consider in vitro culture conditions that better reproduce the joint environment and the growth of biofilms in humans. The null hypothesis of this study was confirmed when no correlation between the antibiotic resistance profile and the biofilm forming ability of the isolated strains was detected.

## Figures and Tables

**Table 1:** Differences in the intensity of biofilm formation for each isolated strain.

INTENSITY OF BIOFILM FORMATION				
ORGANISMS	(HIGH)	(LOW)	(NO)	Total
	n° Strains	n° Strains	n° Strains	n° Strains
*S. epidermidis*	34	16	12	62
*S. aureus*	13	5	4	22
*S. capitis*	6	4	4	14
*S. hominis*	6	5	1	12
*S. haemolyticus*	2	2	-	4
*S. warneri*	-	-	4	4
*M. luteus*	-	-	3	3
*Strep. oralis*	1	1	1	3
*P. avidum*	1	-	1	2
*S. lugdunensis*	2	-	-	2
*S. saprophyticus*	1	-	1	2
*B. fragilis*	-	-	1	1
*B. casei*	1	-	-	1
*C. glucuronolyticum*	1	-	-	1
*M. osloensis*	1	-	-	1
*MRSA*	1	-	-	1
*S. caprae*	-	-	1	1
*S. cohnii*	1	-	-	1
*S. pettenkoferi*	-	-	1	1
*S. simulans*	-	1	-	1
*S. xylosus*	1	-	-	1
**TOTAL**	**72**	**34**	**34**	**140**

**Table 2:** Distribution between colony forming units (CFU) counting and multidrug-resistance (MDR) of the *S. epidermidis* strains. Pearson chi-square *p* = 0.898.

		CFU			Total
		No	Low	High	
**MDR**	**No**	0	2	3	5
	MDR	1	27	29	57
Total		1	29	32	62

**Table 3:** Distribution between colony forming units (CFU) counting and metabolic activity (XTT) of the *S. epidermidis* strains. Pearson chi-square *p* = 0.469.

		CFU			Total
		No	Low	High	
**XTT**	**No**	0	5	3	8
	Low	0	4	10	14
	High	1	20	19	40
Total		1	29	32	62

**Table 4:** Distribution between metabolic activity (XTT) and multidrug-resistance (MDR) of the *S. epidermidis* strains. Pearson chi-square *p* = 0.484.

		XTT			Total
		No	Low	High	
**MDR**	**No**	0	2	3	5
	MDR	8	12	37	57
Total		8	14	40	62

## Supplementary Materials

The following are available online at https://www.mdpi.com/2076-0817/9/8/649/s1, Table S1: Concentration of the antibiotic substances in the discs for the antimicrobial susceptibility tests (BBL™ Sensi-Disc™ Susceptibility Test Discs, BD Life Sciences, Heidelberg, Germany).
