# Supplementary material for: Identification and Morphological Characterization of Biofilms Formed by Strains Causing Infection in Orthopedic Implants
Source: Pathogens. 2020 Aug 12;9(8):649. doi: 10.3390/pathogens9080649 (PMC7460306; doi:10.3390/pathogens9080649)
Supplement: Supplementary file 1 [file pathogens-09-00649-s001.pdf]

Table 1. Concentration of the antibiotic substances in the discs for the antimicrobial susceptibility tests (BBL™ Sensi-Disc™ Susceptibility Test Discs, BD Life Sciences, Heidelberg, Germany).

**Concentration of antibiotic substances in the discs for the antimicrobial susceptibility tests**	
**Antibiotic**	**Concentration**
Amikacin	30 µg
Aminopenicillin	10 µg
Amino + Clav acid	20/10 µg
Azithromycin	15 µg
Aztreonam	30 µg
Cefazolin	30 µg
Cefepime	30 µg
Cefotaxime	30 µg
Cefoxitin	30 µg
Ceftazidime	30 µg
Ceftriaxone	30 µg
Cefuroxime	30 µg
Ciprofloxacin	5 µg
Clindamycin	2 µg
Daptomycin	30 µg
Doripenem	10 µg
Ertapenem	10 µg
Fosfomycin	200 µg
Fusidic acid	10 µg
Gentamicin	10 µg
Imipenem	10 µg
Levofloxacin	5 µg
Linezolid	30 µg
Meropenem	10 µg
Metronizadol	80 µg
Moxifloxacin	5 µg
Mupirocin	5 µg
Penicillin G	1 µg
Piper-Tazob	100/10 µg
Rifampicin	5 µg
Teicoplanin	30 µg
Tetracyclin	30 µg
Tigecyclin	15 µg
Trimeth + Sulf	5 µg
Vancomycin	5 µg
